# Transport stress induces weight loss and heart injury in chicks: disruption of ionic homeostasis via modulating ion transporting ATPases

**DOI:** 10.18632/oncotarget.15903

**Published:** 2017-03-04

**Authors:** Zhao-Yang Li, Jia Lin, Feng Sun, Hui Li, Jun Xia, Xue-Nan Li, Jing Ge, Cong Zhang, Jin-Long Li

**Affiliations:** ^1^ College of Veterinary Medicine, Northeast Agricultural University, Harbin, P. R. China; ^2^ Harbin Sport University, Harbin, P. R. China; ^3^ Department of Heilongjiang for Common Animal Disease Prevention and Treatment, Key Laboratory of the Provincial Education Northeast Agricultural University, Harbin, P. R. China; ^4^ Heilongjiang Key Laboratory for Laboratory Animals and Comparative Medicine, Northeast Agricultural University, Harbin, P. R. China

**Keywords:** transport stress, weight loss, heart injury, ionic disorder, ATPase, Pathology Section

## Abstract

Transportation is inevitable in the poultry industry, and it can induce stress to chicks in varying degrees, such as mild discomfort, sometimes even death. However, the research about the effects of transport stress on the weight loss and heart injury of chicks is lacking. To elucidate the underlying mechanism of transport stress-induced effects, chicks were transported for 2h, 4h and 8h. The creatinine kinase (CK) activities, the ionic contents, the ATPases activities and the transcription of the ATPase associated subunits in chick heart were detected. The results showed that transport stress increased the weight loss and the CK activity, disturbed the ionic (K^+^, Ca^2+^, Mg^2+^) homeostasis and inhibited the ATPase (Na^+^-K^+^-ATPase, Ca^2+^-ATPase, Mg^2+^-ATPase and Ca^2+^-Mg^2+^-ATPase) activities, increased the ATP content and downregulated the gene expression levels of the ATPase associated subunits in heart. In conclusion, transport stress disturbed the ionic homeostasis via modulating ion transporting ATPases and the transcriptions of the associated subunits, and ultimately induced weight loss and heart injury in chicks.

## INTRODUCTION

Stress is a kind of environmental effect on someone, and it may burden its control systems and induce mild discomfort [1]. Animals are often subjected to all kinds of stress in production practice, such as heat stress, cold stress, chemical stress, transport stress and so on. Moreover, transport stress is one of the most common stresses. Transport is inevitable to most of the farmed animals at some period of their lives, sometimes they are transported to another place to be raised, sometimes they are transported to another owner, and sometimes they are transported to slaughter. Together with the social factors (crowding and novel environment, etc.), the physical factors including temperature, noise, a lack of water and feed make the road transport very complicated [2]. So transport stress should be one of the major concerns in animal industry.

Superposition of various stimuli influence animal growth and immunologic function, and cause tissue damage and even death [3]. Previous studies have also reported that transport stress has adverse effects not only on the growth performance, market weight and meat quality of pigs, but also behavioral and biochemical responses of their bodies or peripheral organs of metabolic [4–6]. Mimicking transport stress also induced significant decreases in body weight, disturbance of the organism and significant morphological damage and apoptosis of the intestinal epithelium in rat [7].

Road transport is unavoidable in the poultry industry, and it could cause stress to birds in varying degrees, varying from mild discomfort to death. The effects of the transport on the chicks are associated with various factors, including loading density, temperature, humidity, transit duration and so on [8]. These factors are known to increase body weight loss, as well as the incidence of dead, non-ambulatory, and lame animals during and after the transport [9, 10]. And the transport stress can also induce physiological and metabolic changes [11, 12], which will affect animal welfare, processing yield and meat quality [13–15]. What is worse, it often eventually leads to increased morbidity and mortality, poor meat quality and weight loss, consequently a substantial economic loss [16–18]. It is known to us all, healthy chicks are the starting point for good production performance of poultry, however, the effects of transport stress on chicks are little known.

The previous study has demonstrated that transport stress could cause heart injury in pigs [19], and simulated transport caused damage to the hearts of rats obviously [20]. After being treated with cold stress, apparently the chicken heart tissues were damaged: the cardiac muscle fibers were ruptured, cardiocyte hypertrophy, other heart lesions and histological changes [21]. However, how the transport stress caused heart injury to the chicks is unclear.

As the most important organ and the core of the body, the heart plays an important role in the regulation of blood running and ion homeostasis, which contributes to keeping the weight off. Much evidence has showed that alterations in ion levels may be partially responsible for heart disease [22, 23]. Ionic homeostasis is especially necessary for the heart to function well [24]. Thus, it seems essential and important for cardiac function to maintain ionic homeostasis. However, little is known about the relationship between transport stress and cardiac ionic disorder. Consequently, this present study was aimed to investigate the association between transport stress-induced weight loss, and heart injury, and to assess whether modulation of the cardiac ionic balance is involved in these effects. Finally, we will provide some evidence on the effects of transport stress on chick health.

## RESULTS

### Body weight and biochemical analyses

Changes of weight and creatinine kinase (CK) activity in the control group and transport group were listed in Table 1. Weight loss of the chicks was found after the transport. The body weight decreased to a greater extent in the 2h, 4h, and 8h transport groups compared to control groups and the greatest decrease occurred in the 8h transport group.

**Table 1:** Changes of weight and CK activity of the chicks

Treatment		Weight (g)			CK activity (U/L)
		Before stimulation	After stimulation	Weight loss	
**2h**	**Control**	41.28±3.564	40.70±3.574	0.580	5886.3±24.96
	**Transport**	44.52±3.100	43.32±2.741	1.194	6035.1±339.9
**4h**	**Control**	40.57±2.842	39.76±2.559	0.807	5631.3±76.50
	**Transport**	40.80±3.529	39.98±3.464	0.815	6325.6±312.9*
**8h**	**Control**	42.82±2.255	41.58±2.003	1.235	5760.4±288.2
	**Transport**	43.24±2.871	41.61±2.759	1.628	7105.0±263.9**

Values were expressed as mean ± S.D.. Symbol for the significance of differences between the transport group and control group: *P < 0.05, **P < 0.01 and ***P < 0.001.

To evaluate the degree of transport stress susceptibility in chicks, we firstly investigated the activity of CK in serum, which was the classic index of myocardial injury. As shown in Table 1, when compared with the corresponding control groups, the CK activity increased in all the transport groups and the increase was significant in the 4h and 8h transport groups (*P* < 0.05, *P* < 0.01; Table 1), which indicated that chick heart suffered an injury and continuous stress made it much worse.

### Determination of K^+^ content, the activity of Na^+^-K^+^-ATPase and the transcription of Na^+^-K^+^-ATPase associated subunits

**Figure 1:**
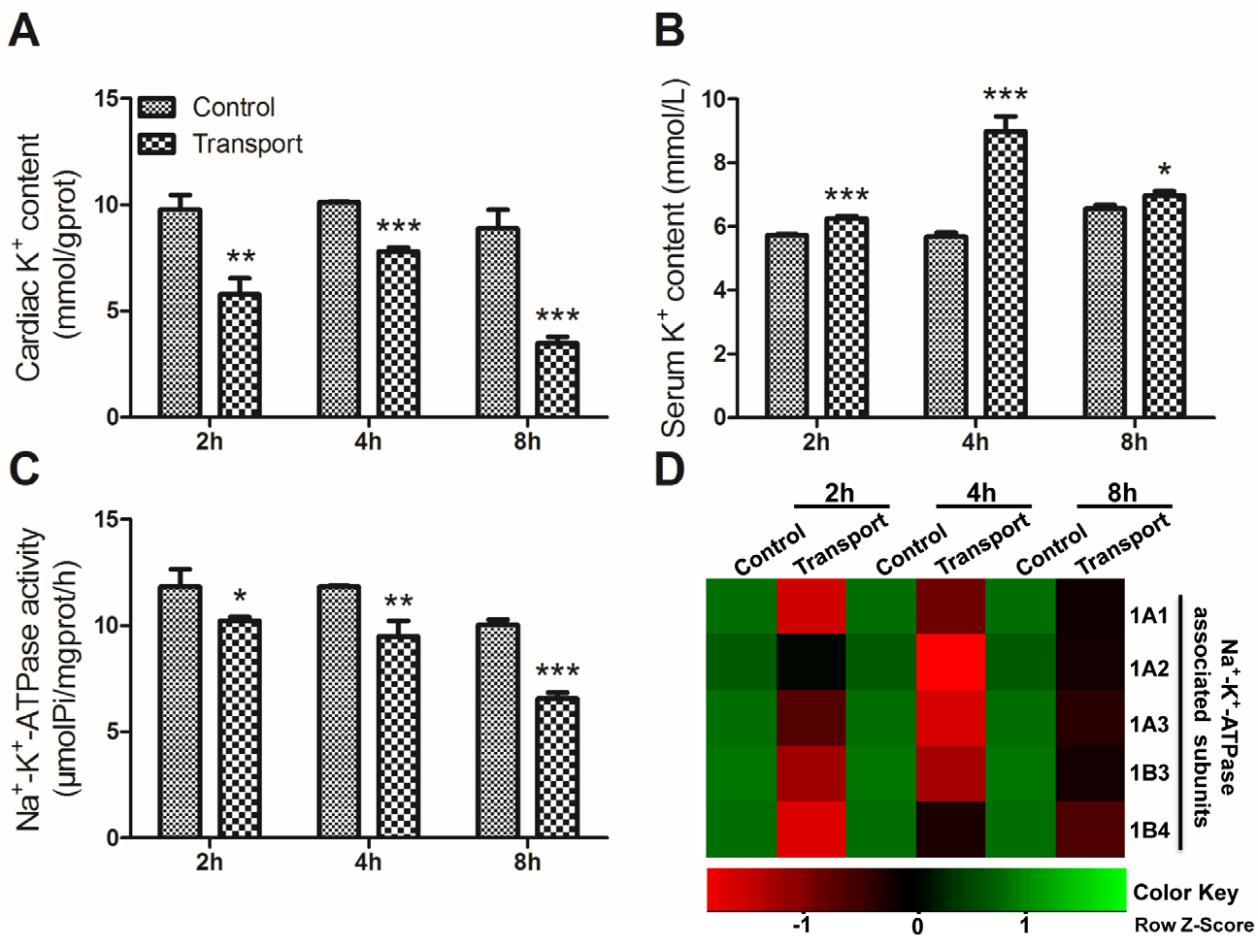
Effects of transport stress on the modulation of K **^+^** transfer channel. **A**. The K^+^ content in cardiac myocytes of the chicks; **B**. The K^+^ content in the serum of the chicks; **C**. The Na^+^-K^+^-ATPase activity in cardiac myocytes of the chicks; **D**. The heat-map of mRNA expression levels of Na^+^-K^+^-ATPase associated subunits in heart. Values were expressed as mean ± S.D.. Symbol for the significance of differences between the transport group and control group: *P < 0.05, **P < 0.01 and ***P < 0.001. The mRNA expression levels of genes transcription are shown using the indicated pseudo color scale from -1 (green) to +1 (red) relative to values for control group. The Row Z-Score represents the relative mRNA expression levels, with the green indicating up-regulated genes, the red indicating down-regulated genes, and black indicating unchanged genes.

The cardiac K^+^ content in all the transport groups was decreased compared with that of control group (*P* < 0.01 or *P* < 0.001; Figure 1A). In comparison with control group, the serum K^+^ content performed significant increase in the 2h, 4h and 8h transport groups (*P* < 0.001 or *P* < 0.05; Figure 1B). Changes of cardiac Na^+^-K^+^-ATPase activities in the transport groups were shown in Figure 1C. The results showed that a notable decrease in Na^+^-K^+^-ATPase activity was found in the 2h transport group (*P* < 0.05) compared to control group, and the decreases were much more significant in the 4h and 8h transport groups (*P* < 0.01, *P* < 0.001) compared to the corresponding control groups, which showed that a longer time of transport led to a much more serious heart injury.

Unsupervised hierarchical clustering of the five Na^+^-K^+^-ATPase subunits mRNA levels showed a unique transcriptional response in the chicks treated with road transport versus the control groups (Figure 1D). It could be easily seen from the results that the transcription of the Na^+^-K^+^-ATPase subunits were down-regulated to some extent after the chicks were transported for 2h, 4h and 8h.

**Figure 2:**
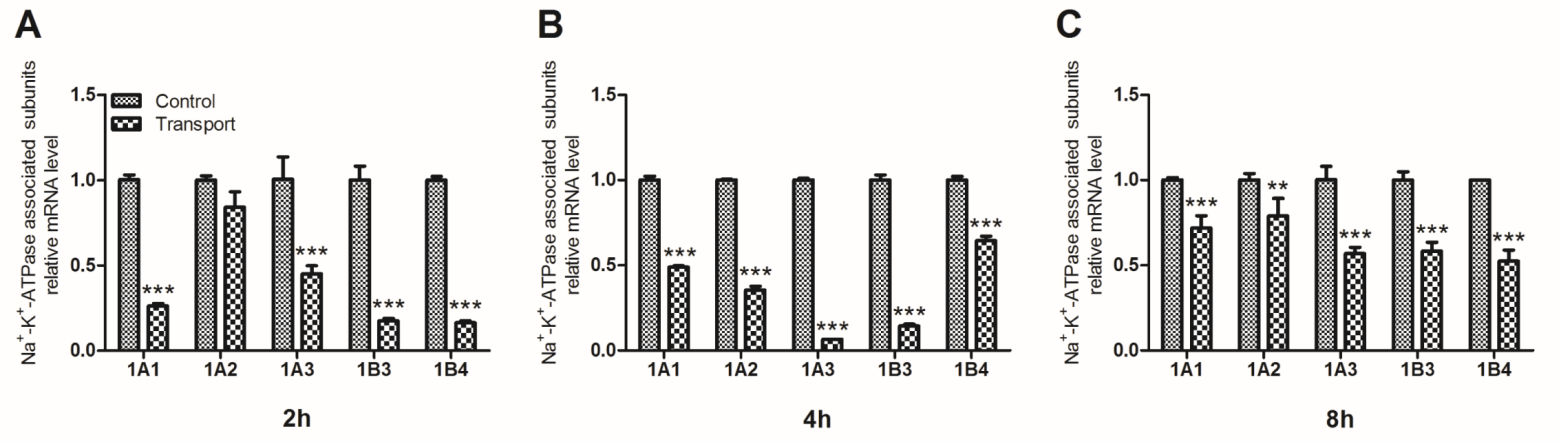
Effects of transport stress on the mRNA expression levels of Na **^+^**-K**^+^**-ATPase associated subunits in chicks heart. **A**. 2h; **B**. 4h; **C**. 8h. Values were expressed as mean ± S.D.. Symbol for the significance of differences between the transport group and control group: *P < 0.05, **P < 0.01 and ***P < 0.001.

The activity of Na^+^-K^+^-ATPase was regulated by its associated subunits. These subunits mRNA levels in chick heart tissue were determined through quantitative real-time quantitative PCR (qRT-PCR) (Figure 2). The data revealed that compared to the 2h control group, the mRNA levels of four subunits (1A1, 1A3, 1B3 and 1B4) substantially decreased in 2h transport group (*P* < 0.001; Figure 2A). And the mRNA expression levels of Na^+^-K^+^-ATPase associated subunits (1A1-1A3, 1B3, 1B4) were also downregulated in the 4h and 8h transport group (*P* < 0.01 or *P* < 0.001), which were statistically different from the corresponding control groups (Figure 2B-2C).

### Determination of Ca^2+^ content, the activity of Ca^2+^-ATPase and the transcription of Ca^2+^-ATPase associated subunits

**Figure 3:**
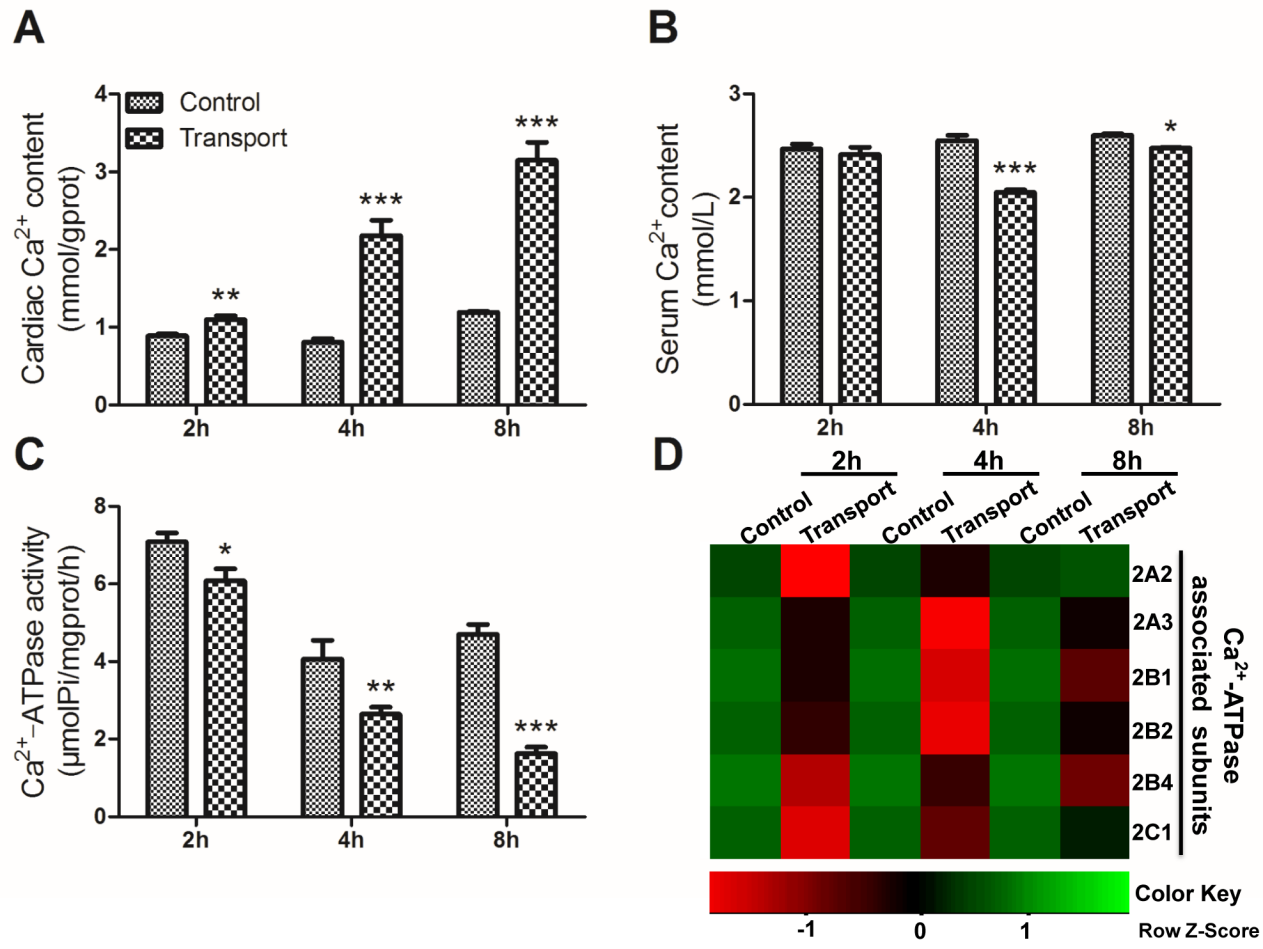
Effects of transport stress on the modulation of Ca **^2+^** transfer channel. **A**. The Ca^2+^ content in cardiac myocytes of the chicks; **B**. The Ca^2+^ content in the serum of the chicks; **C**. The Ca^2+^-ATPase activity in cardiac myocytes of the chicks; **D**. The heat-map of mRNA expression levels of Ca^2+^-ATPase associated subunits in heart. Values were expressed as mean ± S.D.. Symbol for the significance of differences between the transport group and control group: *P < 0.05, **P < 0.01 and ***P < 0.001. The mRNA expression levels of genes transcription are shown using the indicated pseudo color scale from -1 (green) to +1 (red) relative to values for control group. The Row Z-Score represents the relative mRNA expression levels, with the green indicating up-regulated genes, the red indicating down-regulated genes, and black indicating unchanged genes.

Compared to that of the 2h control group, a statistically significant increase in the cardiac Ca^2+^ content was observed in the 2h transport group (*P* < 0.01; Figure 3A), and much more remarkable increases were found in the 4h and 8h transport group (*P* < 0.001; Figure 3A), compared with the corresponding control groups (Figure 3A). In addition, the changes of the serum Ca^2+^ level failed to reach statistical significance in the 2h transport group, while significant decreases occurred in the 4h and 8h transport groups (*P* < 0.001, *P* < 0.05), compared with the corresponding control groups (Figure 3B). The alterations of the cardiac Ca^2+^-ATPase activity were shown in Figure 3C. Significant changes in the cardiac Ca^2+^-ATPase activity were observed in any transport group. The cardiac Ca^2+^-ATPase activity substantially decreased in the 2h transport group (*P* < 0.05) compared to the 2h control group, and the extent of decrease seemed to be much more significant in the 4h and 8h transport groups (*P* < 0.01, *P* < 0.001). More obvious heart damage was observed after the chicks were transported for 4h and 8h.

**Figure 4:**
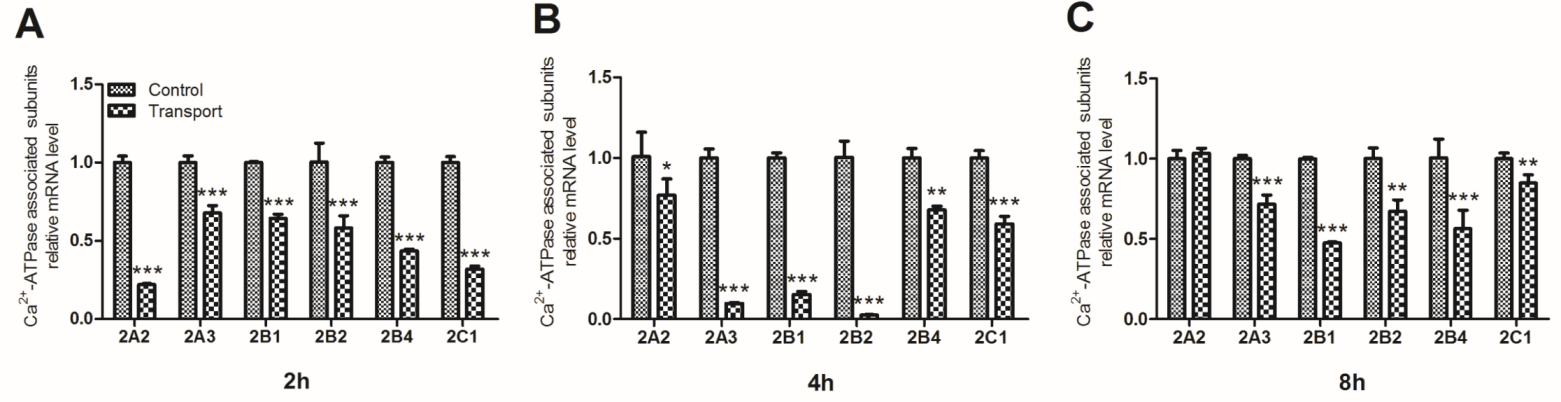
Effects of transport stress on the mRNA expression levels of Ca **^2+^**-ATPase associated subunits in chicks heart. **A**. 2h; **B**. 4h; **C**. 8h. Values were expressed as mean ± S.D.. Symbol for the significance of differences between the transport group and control group: *P < 0.05, **P < 0.01 and ***P < 0.001.

To investigate the unique transcriptional response to transport stress, a heat map showing the mRNA expression levels of the six Ca^2+^-ATPase associated subunits in the transported chicks was presented in Figure 3D. The transcription of the Ca^2+^-ATPase subunits was down-regulated evidently after the chicks were transported for 2h, 4h and 8h. The gene expression levels of the six Ca^2+^-ATPase associated subunits were notablely decreased in the 2h (*P* < 0.001; Figure 4A) and 4h transport groups (*P* < 0.05, *P* < 0.01 or *P* < 0.001; Figure 4B). Additionally, a remarkable decrease of the mRNA expression levels of a set of Ca^2+^-ATPase subunits (2A3, 2B1, 2B2, 2B4 and 2C1) was observed in the 8h transport group.

### Determination of Mg^2+^ content, Mg^2+^-ATPase activity and Ca^2+^-Mg^2+^-ATPase activity

**Figure 5:**
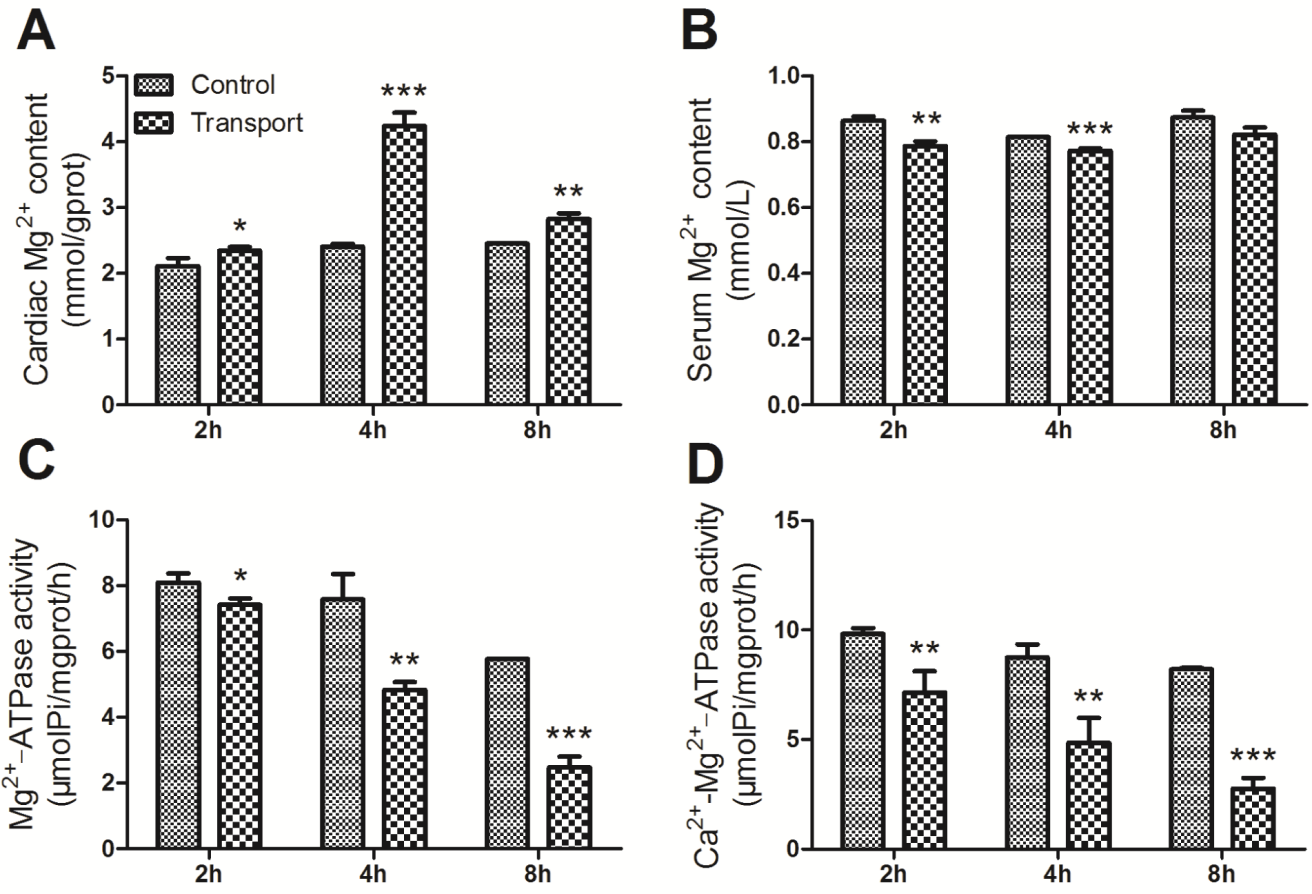
Effects of transport stress on the modulation of Mg **^2+^** transfer channel. **A**. The Mg^2+^ content in cardiac myocytes of the chicks; **B**. The Ca^2+^ content in the serum of the chicks; **C**. The Mg^2+^-ATPase activity in cardiac myocytes of the chicks; **D**. The Ca^2+^-Mg^2+^-ATPase activity in cardiac myocytes of the chicks. Values were expressed as mean ± S.D.. Symbol for the significance of differences between the transport group and control group: *P < 0.05, **P < 0.01 and ***P < 0.001.

Compared with the corresponding control group, the cardiac Mg^2+^ content displayed remarkable increase in all the transport groups (*P* < 0.05, *P* < 0.001, *P* < 0.01), which was shown in Figure 5A. And a statistically significant decrease in the serum Mg^2+^ content was found in the 2h and 4h transport group (*P* < 0.01, *P* < 0.001; Figure 5B) while there was no significant difference in the 8h transport group, compared to that of the corresponding control group. The Figure 5C showed a significant decrease in the cardiac Mg^2+^-ATPase activity in the 2h transport group (*P* < 0.05), and much more remarkable decreases were found in the 4h and 8h transport group (*P* < 0.01, *P* < 0.001). The results of Ca^2+^-Mg^2+^-ATPase activity were presented in Figure 5D. The Ca^2+^-Mg^2+^-ATPase activity in the 2h and 4h transport groups was suppressed significantly (*P* < 0.01, *P* < 0.01), and a more notable decrease was discovered in the 8h transport group compared to the 8h control group (*P* < 0.001).

### Determination of adenosine triphosphate (ATP) content in the heart tissues

**Figure 6:**
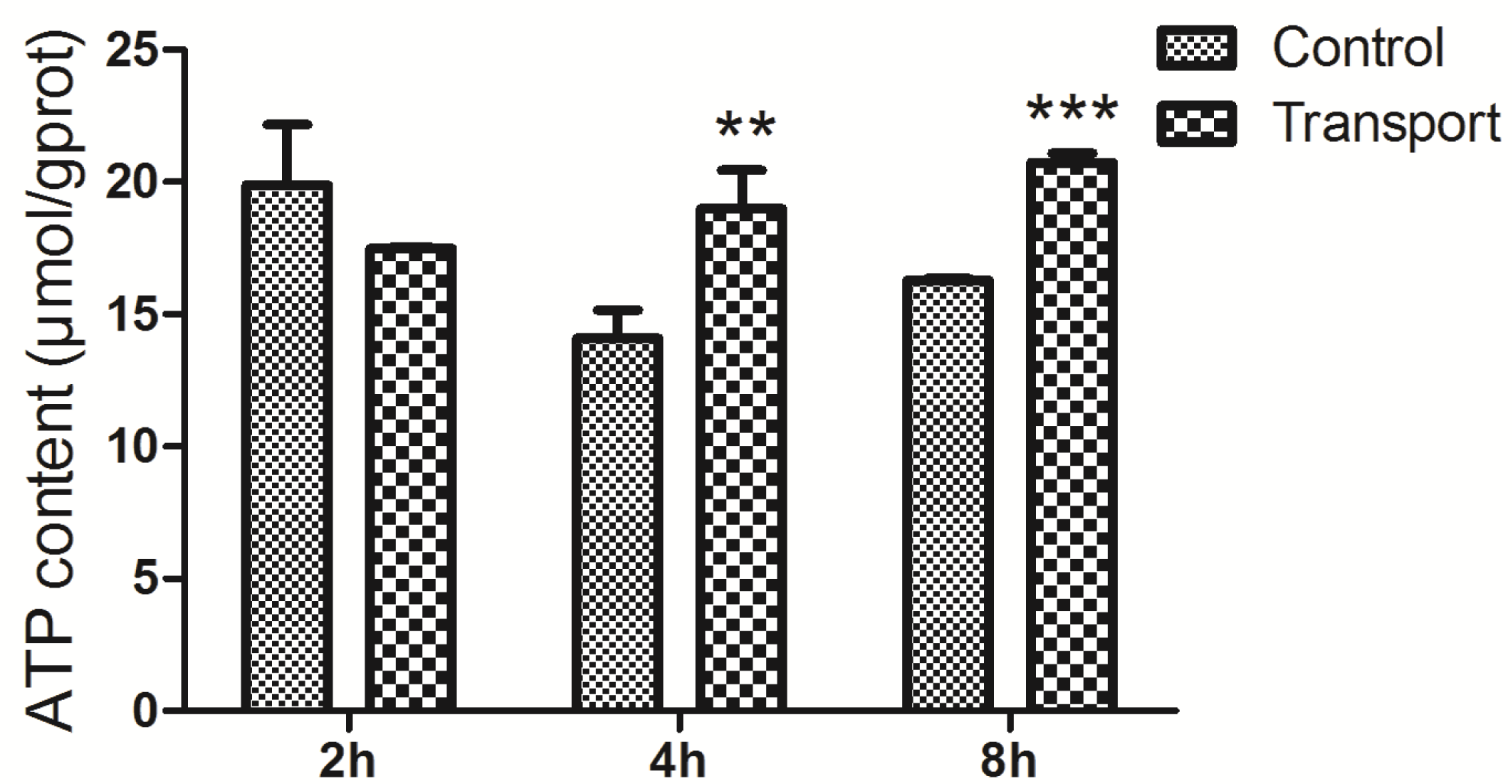
Effects of transport stress on the ATP content in the chicks heart Symbol for the significance of differences between the transport group and control group: *P < 0.05, **P < 0.01 and ***P < 0.001.

The alterations of ATP content were presented in the Figure 6. Significant increases in the ATP content were observed in the 4h (*P* < 0.01) and 8h (*P* < 0.001) transport group compared to that of the corresponding control groups, whereas no notable difference was found in the 2h transport group.

### PCA of cardiac ionic homeostatic modulation

**Figure 7:**
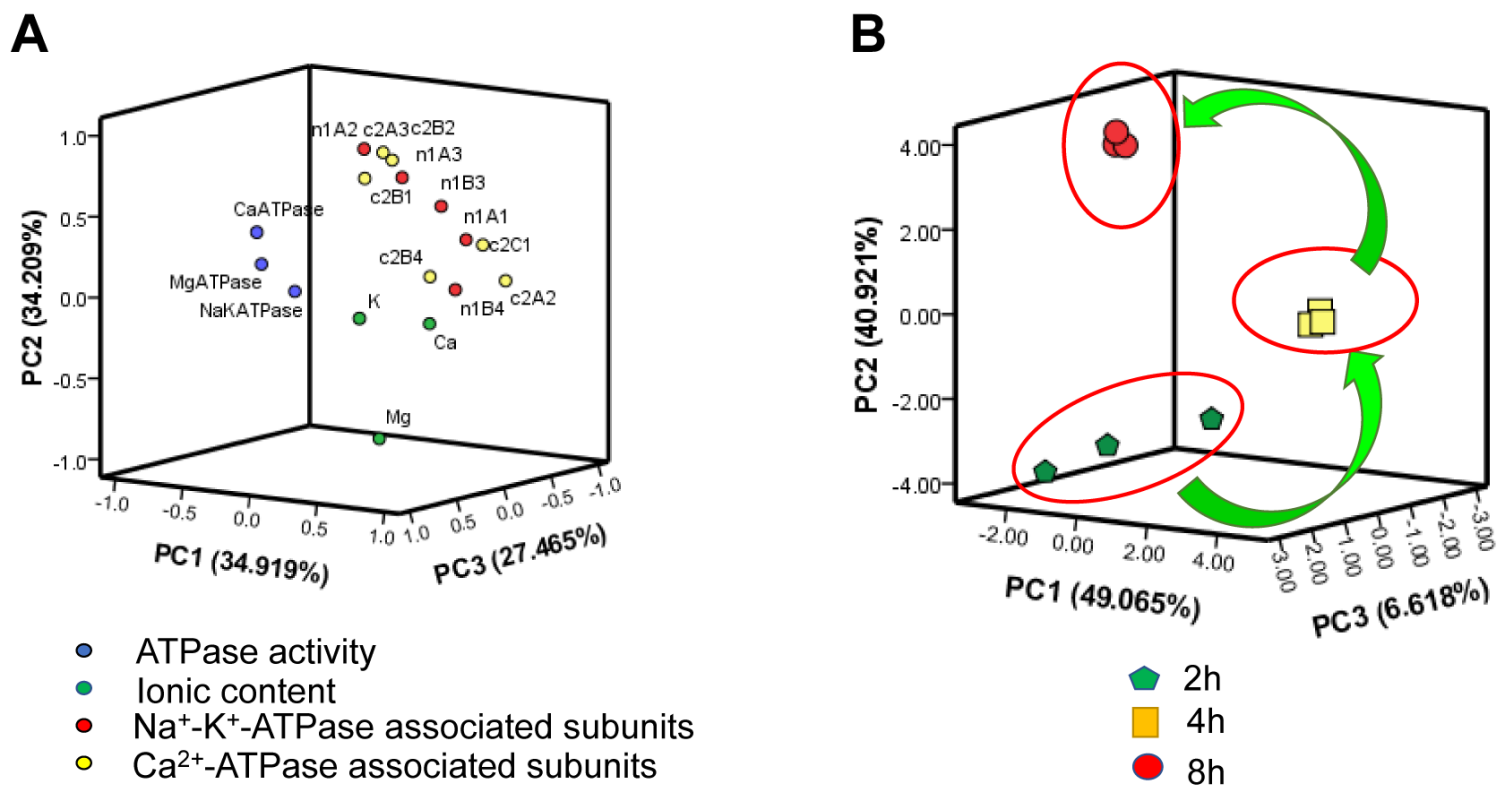
PCA of ionic homeostatic modulation after the transport **A**. Ordination diagram of PCA of biochemical parameters in heart after the transport; **B**. PCA score plot results comparing biochemical parameters at 3 time points.

The information from intercorrelated variables was simplified into some principal components through the linear transformation of original variables by using principal component analysis (PCA). In the present study, all the parameters were divided into the first, second and third principle components based on ordination plots corresponding, and the results were depicted in Figure 7. The sum of PC1, PC2 and PC3 occupied at least 80% of the total variance (Table S2, S3).

The results of PCA of biochemical indices in the heart of chicks subjected to the transport showed that some of the Na^+^-K^+^-ATPase associated genes (1A1, 1B3 and 1B4), Ca^2+^-ATPase associated gene (2A3) might play important roles in response to the heart injury induced by transport stress (Figure 7A). In addition, as an unsupervised pattern recognition method, PCA was used to distinguish potential effects of biochemical parameters in chicks transported for different time periods. The PCA scores revealed a notably time-dependent separation within the transported chicks. Moreover, the changes in metabolic profiles from 2h to 4h and 8h went on in a counterclockwise direction when these changes were obviously separated from the 2h transport group (Figure 7B). That is to say, these results indicated that the changes induced by the transport stress might be time-dependent.

## DISCUSSION

Stress relevant to transport, such as vibration, capture, collision and scrape, heat and cold, thirst and hunger, and fear, has extensive influences on physiological systems in laboratory animals, and changes in the cardiovascular, endocrine, immune, central nervous and reproductive systems will be included [25]. It also reduced the animals’ live weight gain [26, 27] and the quality of animal products [28, 29], and resulted in significant weight loss in emus [30]. The posttransport body weight of all birds was significantly lower than their pretransport body weight [31]. In our present study, a greater weight loss was presented in the road transport treated chicks, which was in agreement with the previous study. Nevertheless, the mechanisms under which transport stress induces live weight loss in chicks are not fully understood.

Heart exerts important function in the regulation of blood running and ion homeostasis, which contributes to keeping the weight off. Previous study has shown that it's possible that transport stress triggers acute heart failure, and this could be one explanation for the sudden death of pigs during the transport [32]. The previous investigations demonstrated that simulated transport stress led to the injury and apoptosis of cardiomyocytes obviously [20]. It has also been reported that road transport could induce tissue damage to the heart [19]. In accordance with the previous studies, our results demonstrated that the CK activities increased significantly after the chicks were subjected to the transport, suggesting an obvious damage to the chick heart. Additionally, in the present study, the transport stress suggested a significant time-dependent effect on the CK activities detected in chick heart.

Proper ionic balance plays a key role in cardiac function [24]. Appropriate ion concentrations are crucial to ensure correct functioning of the entire body, especially the heart. It has been reported that once the ion balance is disrupted, cellular injury and disorders in cardiac function will occur [33, 34]. The changes of cardiac ATPase activities and the transcription of its subunits could disturb the ionic homeostasis and finally induce heart injury in quails [35, 36]. The heat stress caused a loss of ion homeostasis in marine crabs, which impaired the cardiac function [37]. Our findings in this research showed that the ionic homeostasis was disturbed by the transport stress and finally caused damage to the chick heart, which is in accordance with those observations.

As the member of the major category of ion transporters, ATPases are in charge of the basic metabolic and physiological activities of most substances, and they also participate in immediate release of energy. Moreover, they exert important influence in regulating the transmembrane ionic balance to maintain proper function of the vasculature [35, 36]. The previous investigations demonstrated that high temperature inhibited the mitochondrial Ca^2+^-ATPase activity in broilers [38]. And the heat stress could suppress Na^+^-K^+^-ATPase activity and Mg^2+^-ATPase activity of broiler chickens vital organs [39]. The ATPases activities in aquatics, mammals and quails decreased significantly after atrazine exposure [35, 36, 40]. In agreement with these findings, the present study showed that the transport stress caused a damage to the chicks heart and suppressed the activities of Na^+^-K^+^-ATPase, Mg^2+^-ATPase and Ca^2+^-Mg^2+^-ATPase in chicks heart notablely. Additionally, the ATPase activities decreased more significantly when the chicks were transported for more than 2 hours. The results above in the present study indicated that the transport stress disturbed the ion homeostasis by altering the ATPase activities and the damage to the chicks heart was time dependent.

**Figure 8:**
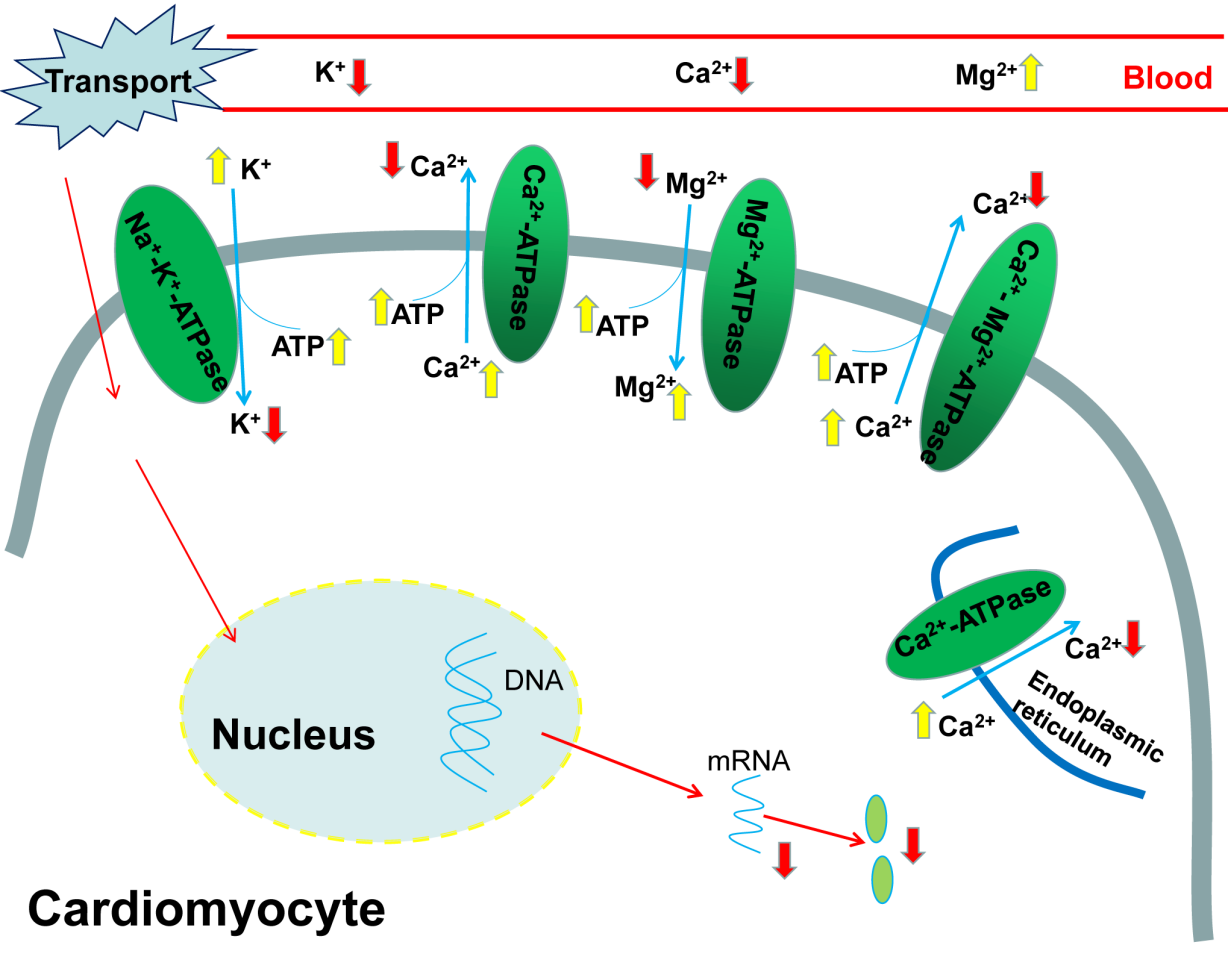
The pathway of transport stress induced ionic disorder in the heart and serum

As the most important one of ionic pumps that control the absorption and secretion of Na^+^ and K^+^, the Na^+^-K^+^-ATPase is responsible for regulating the ion concentration, building and sustaining the transmembranous Na^+^/K^+^ gradient across the cell membrane [41]. As an important part of the plasma membrane, Na^+^-K^+^-ATPase transports 2K^+^ ions into and 3Na^+^ ions out of the cells using 1ATP hydrolysis. Previous investigations have demonstrated that the heat stress significantly reduced Na^+^-K^+^-ATPase in the intestinal mucosa of chickens [39, 42]. The atrazine exposure could induce Na^+^, K^+^ disorders via disruption of Na^+^-K^+^-ATPase [35, 36, 40]. However, to our knowledge, whether the transport stress could influence the Na^+^ and K^+^ contents and cardiac Na^+^-K^+^-ATPase levels of chicks has not been documented so far. In the current study, significant decreases in cardiac K^+^ content, the activity of Na^+^-K^+^-ATPase and the gene expression of Na^+^-K^+^-ATPase associated subunits occurred after the road transport, and remarkable increases in the serum K^+^ content were also found at the same time, which revealed that the K^+^ was transported from the blood into the heart. And our results suggested that the transport stress caused heart damage to the chicks by disturbing the K^+^ homeostasis, and suppressing the Na^+^-K^+^-ATPase activity and subunits transcription (Figure 8).

Ca^2+^ exerts critical function in the excitation-contraction coupling of cardiac myocytes, maintenance of cell integrity and gene expression associated with embryonic heart's growth and development [43, 44]. When the body is in a pathological state such as heart failure, the occurrence of contractile dysfunction and arrhythmias is mainly caused by the mishandling of Ca^2+^ [45]. The previous studies have demonstrated that the heat stress increased the intracellular concentration of free Ca^2+^ [46–48]. In agreement with that, the Ca^2+^ disorders and heart failure were both found in our study. The transport stress resulted in accumulation of calcium in the heart and a decrease in the serum Ca^2+^ contents, which might indicate that the Ca^2+^ was transferred from the blood into the heart. Ca^2+^-ATPase plays a pivotal role in intracellular calcium homeostasis because it can remove calcium from the cytosol across the plasma membrane [49], and calcium accumulation occurrs when it is suppressed substantially. High temperature inhibited the mitochondrial Ca^2+^-ATPase activity and then resulted in increased cytoplasmic Ca^2+^ [38]. The results in the present study also showed that the transport stress induced Ca^2+^ overload and suppressed the Ca^2+^-ATPase activity. Therefore, the observations in our study demonstrated that the transport stress caused the Ca^2+^ disorder by inhibiting the activity of Ca^2+^-ATPase and the gene expressions of Ca^2+^-ATPase associated subunits in the chick heart (Figure 8).

Mg^2+^ plays a vital role in many cellular processes and so many studies have indicated the importance of Mg^2+^ in the etiology of cardiovascular pathology [50]. It has been reported that Mg deficiency enhanced the debilitating effects of ischemic injury [51] and stress sensitivity, which probably resulted in cardiovascular damage and arrhythmias [52]. It can also induce elevation of intracellular Ca^2+^ concentrations, alterations in membrane permeability and transport processes in cardiac cells [50]. A considerable number of enzymes such as Na^+^-K^+^-ATPase and Ca^2+^-ATPase, are dependent on Mg^2+^ directly or indirectly [53–55], and Mg^2+^ is especially important for those enzymes which are of the central importance in the biochemistry of the cell, particularly in energy metabolism. The inhibition of the Mg^2+^-ATPase activity could disturb the Mg^2+^ homeostasis [35]. And the results in our study are in consistent with this: the cardiac Mg^2+^ contents were significantly increased while the serum Mg^2+^ levels decreased evidently. The results in our study suggested that the Mg^2+^ balance was disturbed by the transport stress via suppressing Ca^2+^-Mg^2+^-ATPase activity and Mg^2+^-ATPase activity, which ultimately induced cardiac injury (Figure 8).

ATP is mainly generated in the mitochondria and the mitochondria are responsible for roughly 90% of the energy production of the cell via oxidative phosphorylation [56]. Heart is an excellent source of mitochondria which are able to dynamically supply enough energy to the increased work. This oxidative phenotype could quickly provide a great deal of ATP for cardiac function. It has been reported that mitochondria probably have an outstanding ‘reserve capacity’ and this ‘reserve capacity’ will be consumed when the body suffers severe stress including pressure overload or ischaemia [57, 58]. When subjected to oxidative stress, the cells could utilize ‘reserve capacity’ to increase ATP synthesis via mitochondrial oxidative phosphorylation [59, 60]. Consistent with those, the results in our present study showed that the ATP content in of chicks heart increased significantly in the 4h and 8h transport chicks, which might be a compensatory response to the heart damage. In addition, the downregulation of the ATPase activity may also contribute to the accumulation of ATP. In the present study, the finding showed that the transport induced ionic (K^+^, Ca^2+^, Mg^2+^) disorder in the serum and heart of chicks, the decrease of those ATPase activities, the increase of the ATP content and the downregulation of the ATPase associated subunit expression levels (Figure 8).

In conclusion, the transport stress disturbed the ionic homeostasis via modulating ion transporting ATPases and the transcriptions of the associated subunits, and ultimately induced weight loss and heart injury in chicks. Moreover, long-term transport had a greater negative effect on the chicks. These findings will help to understand how the transport stress caused damage to the chicks and aid the development of guidelines to minimize transport stress in chicks.

## MATERIALS AND METHODS

All procedures, treatments, and animal care were strictly in compliance with the guidelines of the Institutional Animal Care and Use Committee of Northeast Agricultural University (NEAU). The chick's models of transport stress were employed on the basis of Dadgar and Wan's experiments [7, 20, 61]. The study was carried out on clinically healthy chicks weighting about 40.0±3.8g. The animals were obtained from the Xian Feng Chicken Farm in Harbin. Briefly, 120 0-day female chicks (Hy-Line Variety White, Harbin, China) were divided into the Control and Transport groups. Chicks transport crates were employed during transport stress. A separate crate (25°C; relative humidity, 50%) was used for each replicate group: transport group was transported by road for 2h, 4h or 8h over a distance about 480 km with an average speed of 60 km/h and the control group was not subjected to road transport. Feed and water were not available to the chicks during the transport periods. 20 chicks in each group were euthanized with carbon dioxide after treatment. Then, they were slaughtered immediately via exsanguinations of the jugular vein with bistouries and the heart tissues from each chick were collected. Whole blood was centrifuged at 2500g for 10 mins to obtain serum. Remove the heart tissues from the chicks at once on an ice bag and wash them thoroughly with physiological saline solution. Then both tissues and serum were preserved at -80°C for subsequent tests. Before and after the experiment, all the animals were weighed.

### Biochemical assays

Serum CK activities have been taken as important indexes of heart injury. CK activity in the serum was detected by RT-9200 semi-automatic biochemical analyzer (Rayto Life and Analytical Sciences Co., Ltd. China), using the performance rate method.

### Determination of protein content

Protein content was determined by the dye-binding method of Bradford. The standard curve was constructed by using Bovine serum albumin (BSA).

### Determination of the K^+^, Ca^2+^ and Mg^2+^ concentrations in the heart and serum

About one gram of heart tissue was minced and homogenized (ten times the weight of tissue) in physiological saline and then centrifuged (3,000 g for 10 mins). The resulting clear supernatant was collected for ionic content and ATPase activity estimations. The cardiac K^+^, Ca^2+^ and Mg^2+^ concentrations and the serum K^+^, Ca^2+^ and Mg^2+^ concentrations were determined by using the detection kits (Nanjing Jiancheng Bioengineering Institute, China).

### Cardiac ATPase activity assays

The assay kits (Nanjing Jiancheng Bioengineering Institute, China) were used to mensurate the activities of Na^+^-K^+^-ATPase, Ca^2+^-ATPase, Mg^2+^-ATPase and Ca^2+^-Mg^2+^-ATPase in heart tissue. And the determinations of these ATPases were independent of each other, which meant the test of one ATPase couldn't be influenced by another.

### Total RNA isolation and qRT-PCR

A phenol and guanidine isothiocyanate-based TRIzol reagent (Beijing Tiandi, Inc. China) was used to extract Total RNA from the heart tissue following the instructions. A spectrophotometer was used to assess the concentration and purity based on OD280/OD260 and OD260/OD230 ratios, respectively. The primers for qRT-PCR were designed using Oligo 7.22 Software (Molecular Biology Insights, Cascade, CO) and the primer sequences were shown in Table S1. qRT-PCR was performed using LightCycler^®^ 480 Real-Time PCR System (Roche, CH). The Lyceraldehyde-3-phosphate dehydrogenase (GAPDH) and β-actin were used as housekeeping controls to normalize the amounts of cDNA among the samples. The mRNA relative abundance was calculated according to the 2^−ΔΔCT^ method [62] and the results were normalized to the mean of GAPDH and β-actin.

### Statistical analysis

The data were presented as the mean ± standard deviation (S.D.) and were analyzed using GraphPad Prism5.0 (GraphPad Software, USA) and SPSS 17.0 software (SPSS Inc., USA). One-way ANOVA followed by Tukey's post hoc pairwise comparison was used to perform the statistical analyses. Asterisks (*) indicate statistically significant differences from the control group, *P < 0.05, **P < 0.01 and ***P < 0.001. Ranking of genes by degree of differential expression was analyzed with a heat map using the R Programming Language version 3.2.1. Additionally, PCA can be used to effectively simplify the information from intercorrelated into some principal components variables through linear transformation of the original variables, and it was performed in the present study to determine the foremost parameters. Then these parameters would be used as pivotal factors for individual variations using the same software. In accordance with the Spearman's test, the observed relationships among the parameters could be confirmed and quantified.

## SUPPLEMENTARY MATERIALS TABLES


