# Supplementary material for: Transport stress induces weight loss and heart injury in chicks: disruption of ionic homeostasis via modulating ion transporting ATPases
Source: Oncotarget. 2017 Mar 4;8(15):24142–53. doi: 10.18632/oncotarget.15903 (PMC5421834; doi:10.18632/oncotarget.15903)
Supplement: Supplementary file 1 [file oncotarget-08-24142-s001.pdf]

Transport stress induces weight loss and heart injury in chicks: disruption of ionic homeostasis via modulating ion transporting ATPases

Supplementary Material

Table S1. Oligonucleotides used in the present study.

	Gene	Forward Primer（5'→3'）	Reverse Primer（5'→3'）	Product length	NCBI accession No.
House keeper	**GAPDH**	CTTTGGCATTGTGGAGGGTC	ACGCTGGGATGATGTTCTGG	128 bp	NM_204305.1
	**β-actin**	AGTACCCCATTGAACACGGC	CTCCTCAGGGGCTACTCTCA	98 bp	NM_205518.1
Na^+-K^+-ATPase associated subunits	**1A1**	TACAGCCTTCTTTGTCAGCA	CAGCTAGAGCAGTCTCCTCGAA	140 bp	NM_205521.1
	**1A2**	GCCGCTGTCGTCATTGTCA	TCTCCGCGTTGATTTGGAT	142 bp	NM_205476.1
	**1A3**	CCATCCTCAAGCGTGACGTG	ACTTTGACGGACCCGGACGA	85 bp	NM_205475.1
	**1B3**	GCAGTTCGTCTACAACCCCA	TAAGCCCTGGACTAGAAATCCG	194 bp	NM_205535.1
	**1B4**	GCCAGGAGTAATGATCAGACCA	TCATCGTAAGCTGCTAGGAAC	120 bp	NM_001044651.1
Ca^{2+}-ATPase associated subunits	**2A2**	GCGATTTGCTTGTCCATGTCAC	ATCAGCCATTGCGTCACGTT	101 bp	NM_001271973.1
	**2A3**	ACATCCGCATCATTGAAATCCG	ACCCCTGTTGCAATGACGAT	199 bp	NM_204891.1
	**2B1**	GAAACAAAACTGAATGTGCCTTGCT	GCAACTTCTCTTCTGGTATCTCATTCCGTA	93bp	NM_001168002.3
	**2B2**	CTGCCATCCTGCTGTCTGTTATCTGTG	CCACGAAACTGCTTCTCCTTGCTCC	79bp	XM_015293479.1
	**2B4**	AGAAAGCCAAGACGTTCC	AATTCATTGTCGCCACCT	130bp	XM_015298965.1
	**2C1**	TTGCCTTTGTTCAGGAGTACCGAT	TTCCACTCTACCTTCTCGCACAC	98bp	XM_015281760.1

Table S2. Total Variance Explained of PCA for Figure 7A

Total Variance Explained

Component	Initial Eigenvalues			Extraction Sums of Squared Loadings			Rotation Sums of Squared Loadings		
	Total	% of Variance	Cumulative %	Total	% of Variance	Cumulative %	Total	% of Variance	Cumulative %
1	10.636	62.562	62.562	10.636	62.562	62.562	5.936	34.919	34.919
2	3.629	21.348	83.910	3.629	21.348	83.910	5.815	34.209	69.127
3	2.156	12.682	96.592	2.156	12.682	96.592	4.669	27.465	96.592
4	.193	1.138	97.730						
5	.129	.760	98.490						
6	.075	.441	98.931						
7	.055	.322	99.253						
8	.045	.263	99.516						
9	.036	.211	99.727						
10	.016	.095	99.822						
11	.011	.066	99.888						
12	.008	.048	99.936						
13	.005	.029	99.965						
14	.003	.016	99.980						
15	.002	.014	99.995						
16	.001	.004	99.999						
17	.000	.001	100.000						

Extraction Method: Principal Component Analysis.

Table S3. Total Variance Explained of PCA for Figure 7B.

Total Variance Explained

Component	Initial Eigenvalues			Extraction Sums of Squared Loadings			Rotation Sums of Squared Loadings		
	Total	% of Variance	Cumulative %	Total	% of Variance	Cumulative %	Total	% of Variance	Cumulative %
1	9.924	49.619	49.619	9.924	49.619	49.619	9.813	49.065	49.065
2	8.145	40.725	90.343	8.145	40.725	90.343	8.184	40.921	89.985
3	1.252	6.260	96.604	1.252	6.260	96.604	1.324	6.618	96.604
4	.365	1.826	98.430						
5	.183	.914	99.344						
6	.058	.288	99.632						
7	.045	.223	99.855						
8	.029	.145	100.000						
9	3.455E-16	1.727E-15	100.000						
10	3.248E-16	1.624E-15	100.000						
11	2.725E-16	1.363E-15	100.000						
12	2.053E-16	1.026E-15	100.000						
13	1.144E-16	5.718E-16	100.000						
14	8.443E-17	4.222E-16	100.000						
15	-3.947E-17	-1.973E-16	100.000						
16	-9.499E-17	-4.750E-16	100.000						
17	-2.070E-16	-1.035E-15	100.000						
18	-3.262E-16	-1.631E-15	100.000						
19	-4.144E-16	-2.072E-15	100.000						
20	-4.729E-16	-2.364E-15	100.000						

Extraction Method: Principal Component Analysis.
